# Enhanced BMP-2-Mediated Bone Repair Using an Anisotropic Silk Fibroin Scaffold Coated with Bone-like Apatite

**DOI:** 10.3390/ijms23010283

**Published:** 2021-12-28

**Authors:** Christian Deininger, Andrea Wagner, Patrick Heimel, Elias Salzer, Xavier Monforte Vila, Nadja Weißenbacher, Johannes Grillari, Heinz Redl, Florian Wichlas, Thomas Freude, Herbert Tempfer, Andreas Herbert Teuschl-Woller, Andreas Traweger

**Affiliations:** 1 Institute of Tendon and Bone Regeneration, Spinal Cord Injury & Tissue Regeneration Center Salzburg, 5020 Salzburg, Austria; c.deininger@salk.at (C.D.); andrea.wagner@pmu.ac.at (A.W.); nadja.weissenbacher@pmu.ac.at (N.W.); herbert.tempfer@pmu.ac.at (H.T.); 2 Department of Orthopedics and Traumatology, Salzburg University Hospital, Paracelsus Medical University, 5020 Salzburg, Austria; f.wichlas@salk.at (F.W.); t.freude@salk.at (T.F.); 3 Austrian Cluster for Tissue Regeneration, 1200 Vienna, Austria; patrick.heimel@trauma.lbg.ac.at (P.H.); e.salzer@tue.nl (E.S.); monforte@technikum-wien.at (X.M.V.); johannes.grillari@trauma.lbg.ac.at (J.G.); heinz.redl@trauma.lbg.ac.at (H.R.); 4 AUVA Research Centre, Ludwig Boltzmann Institute for Experimental and Clinical Traumatology, 1200 Vienna, Austria; 5 Karl Donath Laboratory for Hard Tissue and Biomaterial Research, University Clinic of Dentistry, Medical University of Vienna, 1090 Vienna, Austria; 6 Department Life Science Engineering, University of Applied Sciences Technikum Wien, 1200 Vienna, Austria; 7 Department of Biotechnology, Institute of Molecular Biotechnology, BOKU-University of Natural Resources and Life Sciences, 1180 Vienna, Austria

**Keywords:** silk scaffold, pseudarthrosis, nonunion, critical sized defect, bone regeneration

## Abstract

The repair of large bone defects remains challenging and often requires graft material due to limited availability of autologous bone. In clinical settings, collagen sponges loaded with excessive amounts of bone morphogenetic protein 2 (rhBMP-2) are occasionally used for the treatment of bone non-unions, increasing the risk of adverse events. Therefore, strategies to reduce rhBMP-2 dosage are desirable. Silk scaffolds show great promise due to their favorable biocompatibility and their utility for various biofabrication methods. For this study, we generated silk scaffolds with axially aligned pores, which were subsequently treated with 10× simulated body fluid (SBF) to generate an apatitic calcium phosphate coating. Using a rat femoral critical sized defect model (CSD) we evaluated if the resulting scaffold allows the reduction of BMP-2 dosage to promote efficient bone repair by providing appropriate guidance cues. Highly porous, anisotropic silk scaffolds were produced, demonstrating good cytocompatibility in vitro and treatment with 10× SBF resulted in efficient surface coating. In vivo, the coated silk scaffolds loaded with a low dose of rhBMP-2 demonstrated significantly improved bone regeneration when compared to the unmineralized scaffold. Overall, our findings show that this simple and cost-efficient technique yields scaffolds that enhance rhBMP-2 mediated bone healing.

## 1. Introduction

While adult bone generally heals without complication, resulting in full recovery of bone tissue and function, approximately 10% of all fractures do not heal adequately and appropriate stability is not achieved. Risk factors including open fractures, co-morbidities, large osseous defects after trauma, prosthesis loosening, or extended tumor resection can, however, preclude full tissue regeneration, ultimately resulting in a delayed union, non-union, or malunion [1,2]. In case of an atrophic or hypotrophic pseudarthrosis, vitalizing of the bony edges and implantation of a cancellous bone graft, e.g., of the iliac crest, the proximal tibia or from the calcaneus, is generally considered as gold standard. Alternatively, autologous intramedullary bone grafts can also be harvested from the femoral shaft using the Reamer-irrigator aspirator device (RIA) [3,4,5]. However, supply is often limited, and these treatment options have demonstrated varied success rates. Further, they can be associated with donor site morbidity such as persisting pain or, in rare cases, fractures of the pelvis [6]. Therefore, the use of allografts or synthetic biomaterials [7,8] have been developed to overcome these limitations. However, these substitute materials often lack osteogenic properties [9].

Recombinant human bone morphogenetic protein 2 (rhBMP-2) is currently the only FDA-approved osteoinductive growth factor in clinical use to improve bone repair. However, rhBMP-2-associated adverse events, e.g., ectopic bone formation, inflammation, and osteolysis have been reported [10]. The majority of the complications are believed to arise due to the use of excessive, supraphysiological concentrations of rhBMP-2. Therefore, scaffold-based strategies to improve BMP-2-mediated bone regeneration have the potential to minimize the occurrence of adverse events and make the use of rhBMP-2 safer and more cost-effective.

Silk fibroin is characterized by its good biocompatibility and tunable mechanical properties [11,12,13]. Due to the remarkable versatility for scaffolding, silk fibroin allows the manufacture of bone substitute material with an optimized architecture. For example, pore sizes ranging from 100 to 350 µm have been shown to promote bone formation [14]. In addition to optimal porosity, scaffold anisotropy has been shown to promote bone repair [15], potentially by enabling guided cell migration into the defect area [16]. Anisotropic biopolymer scaffolds can be produced via directed ice-templating techniques, yielding constructs with axially aligned pores [17]. Next to the scaffold micro- and macroarchitecture, simple and cost-effective biomimetic coatings of orthopedic implants using simulated body fluid (SBF) solutions can result in improved biocompatibility and bone regeneration [18]. Therefore, the aim of this study was to evaluate the bone regenerative capacity of an anisotropic, highly porous silk scaffold coated with bone-like apatitic calcium phosphate in combination with a low dose of rhBMP-2 in a critical-sized defect model (CSD) of the rat femur.

## 2. Results

### 2.1. Generation of Bone-Mimetic Silk Scaffolds

**Figure 1:**
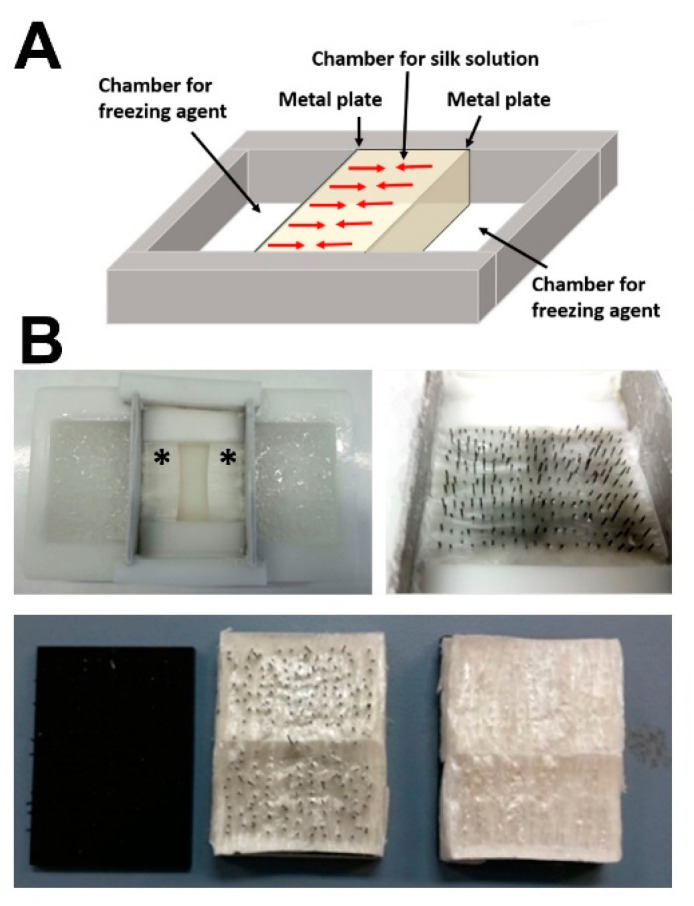
(**A**) Schematic representation of the directional freezing process for the production of anisotropic silk scaffolds; red arrows indicate the freezing direction starting from the two cooled down metal plates (**B**) the freezing process yielded a reproducible freezing front (*) and interconnectivity of the resulting axial pores was ensured by placing a needle array perpendicularly within the silk fibroin solution during freezing (top right), which was removed from the resulting silk scaffold after freeze-drying (bottom).

Silk fibroin scaffolds were generated using a unidirectional freeze-drying process (Figure 1A) using a custom-made mold (Figure 1B). Pore interconnectivity was achieved by placing an array with polypropylene rods (spacing 3 mm; diameter 300 µm) within the freezing silk fibroin solution and subsequent removal of the array after freeze-drying (Figure 1B, bottom).

**Figure 2:**
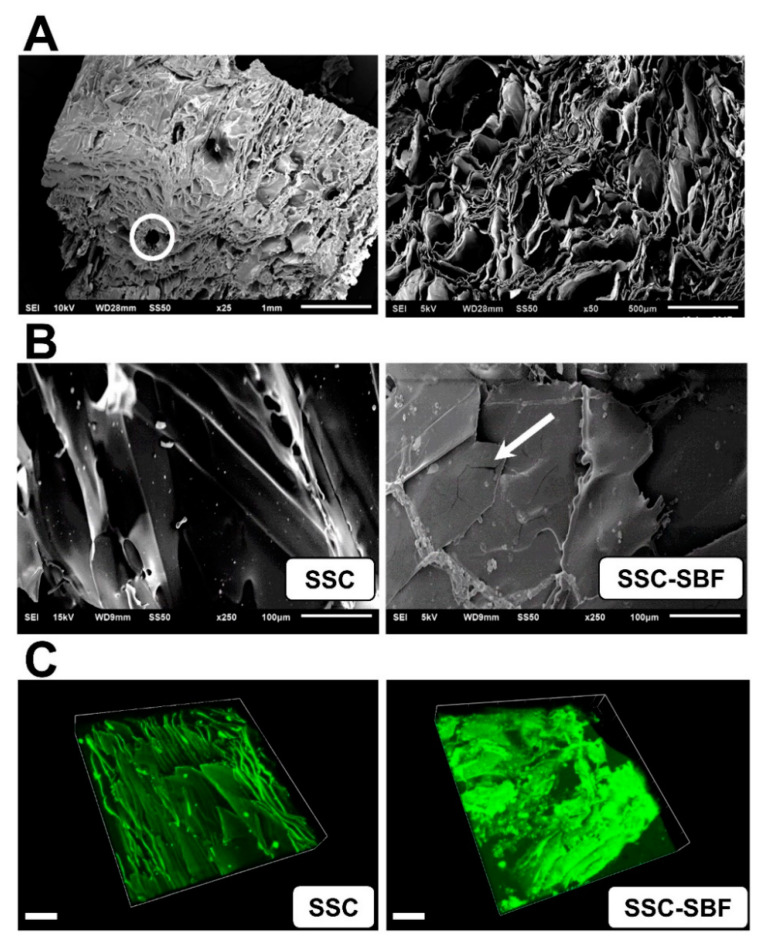
(**A**) Scanning electron micrograph (SEM) showing the highly aligned, unidirectional channel-like pores and transverse interconnecting channels produced by the needle array (white circle); (**B**) SEM image of an untreated silk scaffold (SSC; left image) and a SSC after treatment with 10× simulated body fluid (SSC-SBF; right image), demonstrating a uniform coating of the scaffold with intermittent aggregates and cracks within the coating (arrow). (**C**) Laser scanning confocal microscopy image demonstrating a significant increase in autofluorescence (ex: 488 nm; em: 518 nm) after treatment of an SSC with 10× SBF (SSC-SBF), indicating uniform surface mineralization (Scale bar = 100 µm).

Scanning electron microscopy (SEM) analysis of the native silk fibroin scaffolds (SSC) revealed a highly porous structure, with axially aligned channel-like pores (Figure 2A). After treatment with 10× SBF (SSC-SBF), surface mineralization was observed by SEM, revealing a thin, evenly distributed coating with occasional crack-like structures (Figure 2B). This was further corroborated by laser confocal fluorescence imaging, demonstrating an even coating interspersed with granules as demonstrated by a significant increase in autofluorescence (ex: 488 nm; em: 518 nm) signal intensity after SBF-treatment (Figure 2C).

**Figure 3:**
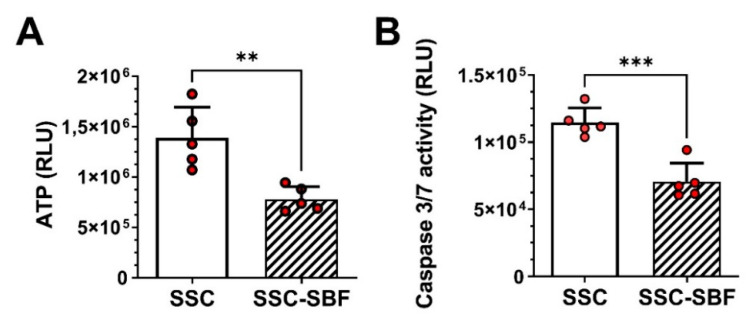
(**A**) ATP content and (**B**) caspase 3/7 activity of rBMSCs seeded onto untreated (SSC) and 10× SBF-treated silk scaffolds (SSC-SBF). Results are shown as the mean of five independent replicates ± SD (** *p* < 0.01, *** *p* < 0.001; unpaired *t*-test).

To evaluate any cytotoxic effects, SSC and SSC-SBF scaffolds were statically seeded with 15000 rat bone marrow-derived mesenchymal stromal cells (rBMSCs) and after 3 days in culture cellular ATP content was measured as a surrogate of cell viability. Further, caspase 3/7 activity was determined to evaluate signs of early apoptosis. ATP content was lower in the mineralized silk scaffolds when compared to the untreated scaffolds (Figure 3A), whereas caspase 3/7 activity was relatively higher in the untreated scaffolds (Figure 3B). However, when calculating the ratio of ATP production to caspase 3/7 activity based on the RLU (relative light units), no difference between the SSC and SSC-SBF scaffolds was obvious (12.20 ± 2.92 vs. 11.40 ± 2.82; *p* = 0.67, unpaired *t*-test).

### 2.2. General Animal Health

**Figure 4:**
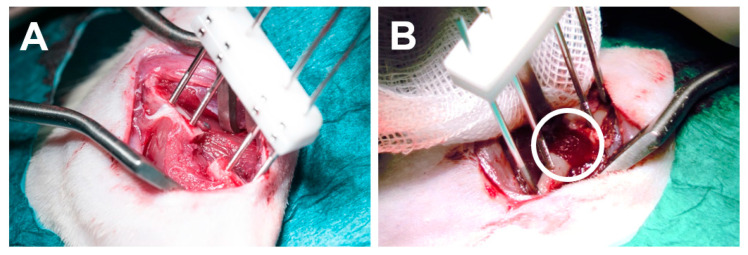
(**A**) Intraoperative view of the critical sized defect in the right femur before and after (**B**) placement of the silk scaffold.

In total, 24 rats were included in the study. No intraoperative complications were recorded for the animals included and the surgery was well tolerated. rhBMP-2-loaded and unloaded SSC or SSC-SBF scaffolds were press-fit into a 5 mm CSD of the right femur, resulting in a stable placement of the scaffold within the defect. (Figure 4A,B). All animals showed normal behavior, with full weight bearing 48 h after surgery. However, during the X-ray evaluations 1 animal each in the SSC, SSC-SBF, and SSC-SBF+BMP-2 group showed signs of osteolysis and loosening of the internal fixator. Therefore, these animals were euthanized prior to the endpoint of the study.

### 2.3. X-ray, µCT Analysis, and Descriptive Histology 10 Weeks Post-Surgery

**Figure 5:**
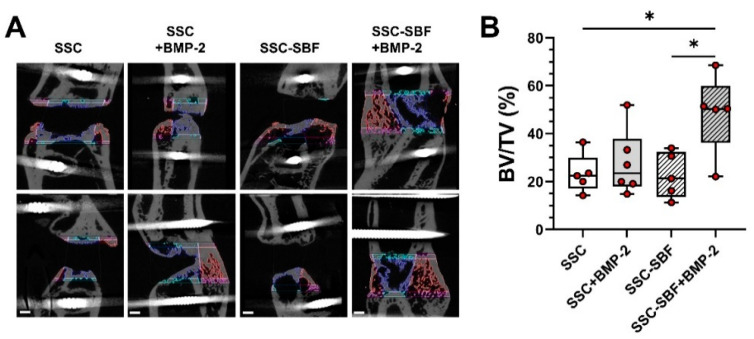
(**A**) μCT images in two planes showing the defect region for a representative specimen of each treatment group (10 weeks after surgery). The color-coded regions delineate the regions included for quantification of the bone volume (blue: defect bone volume; red: bone callus volume; pink and turquoise: bone margins; scale bar = 1 mm); (**B**) BV/TV measurements determined for the different treatment groups 10 weeks after treatment (*n* = 5–6); * *p* < 0.05 (one-way ANOVA).

**Figure 6:**
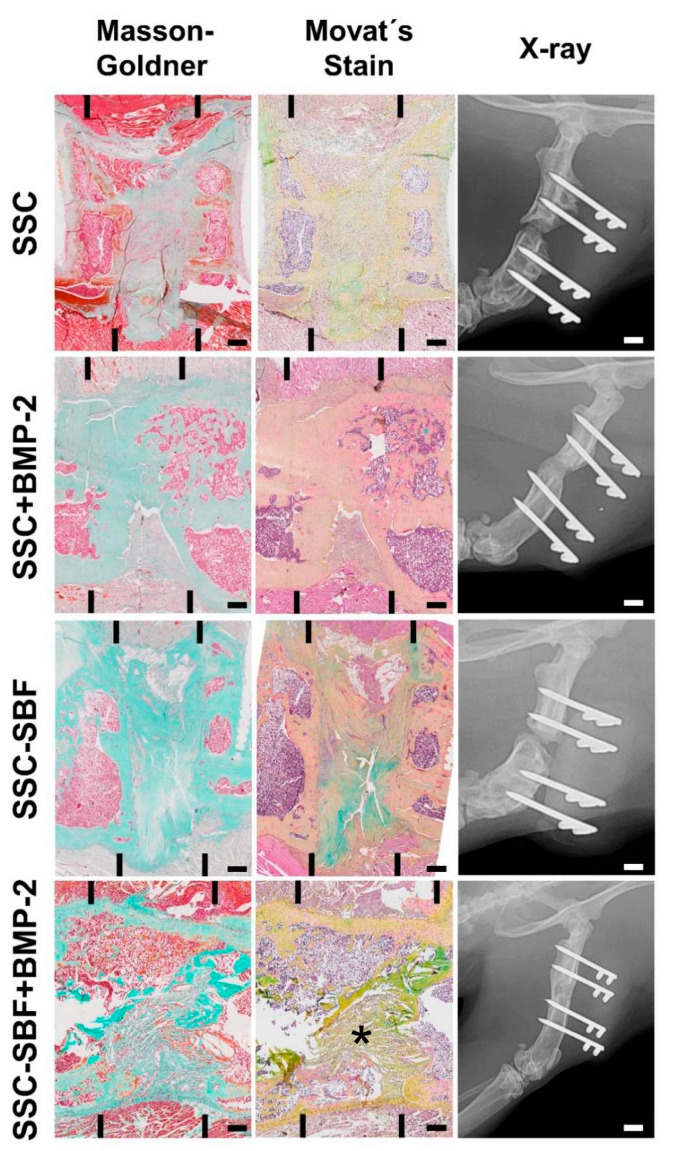
Representative histological sections of the defect area for all treatment groups stained either with Masson–Goldner trichrome (**left**) or Movat’s pentachrome (**middle**) stain. The margins of the original defect are indicated by black bars (Scale bar = 1 mm). The **right** column shows X-rays of the same samples in a lateral plane 10 weeks postoperatively with the internal fixator still in place (Scale bar = 4.5 mm). * remnants of silk fibroin scaffold.

Newly formed bone tissue was quantified using microcomputed tomography (μCT). Comparisons of the newly formed bone tissue (BV/TV; Figure 5A,B) 10 weeks after surgery revealed a significant difference (*p* < 0.05; one-way ANOVA) between the SSC-SBF+BMP-2 (48.50% ± 16.7%) and the SSC (23.31% ± 8.2%) as well as the SSC-SBF treated group (22.7% ± 9.5%). Treatment with SSC-BMP-2 resulted only in a moderate increase in bone volume (27.67% ± 13.6%). In comparison, defects treated with an absorbable collagen sponge (ACS) revealed moderate to no bone formation (16.67 ± 16.28%; Figure S1A–C). Radiographic examination (Figure 6) revealed 5 of the 21 femoral defects showing bony bridging; 1 out of 6 within the SSC+BMP-2 group (16.7%) and 4 out of 5 after treatment with SSC-SBF+BMP-2 (80.0%). In addition, a haptic test for “bending stability” was performed in a blinded fashion, further corroborating the findings by X-ray as only the 5 healed femora were identified as stable by 2 independent examiners. 

New bone deposition identified by descriptive histology using Masson–Goldner trichrome staining (Figure 6 left row) or Movat’s pentachrome staining (Figure 6 middle row) matched the mineral depositions visualized by μCT. Minimal bone regeneration was seen in defects treated with SSC, SSC-SBF (Figure 6), or ACS (Figure S1D). However, there was no cortical bridging obvious and the defect gap was predominantly filled with fibrotic tissue. In addition, the majority of the samples showed closure of the medullary canal (“capping”). In contrast, defects treated with SSC+BMP-2 showed moderately more newly formed bone tissue. Cortical bridging, however, was only evident in 1 sample (shown in Figure 6). Overall, the extent of bone and bone marrow formation was greatest in the CSDs treated with SSC-SBF+BMP-2, resulting in cortical bridging in 4 out of 5 samples. Generally, the silk fibroin scaffolds were resorbed within 10 weeks after implantation. Only occasionally, remnants of the scaffold were evident (indicated by star in Figure 6, bottom row).

## 3. Discussion

The goal of the present study was to develop a biomimetic silk fibroin scaffold which enhances rhBMP-2-mediated regeneration of critical sized segmental bone defects. By using a simple and cost-effective method we generated silk fibroin scaffolds with anisotropic channel-like pores extending throughout the scaffold which were then coated with bone-like apatitic calcium phosphate by immersion in 10× SBF. Significant differences in bone healing were evident in vivo between the various treatment groups, with superior performance of the SSC-SBF scaffold loaded with a low dose of rhBMP-2 (2.5 µg). In contrast, the uncoated, anisotropic silk fibroin scaffold loaded with a similar dose of rhBMP-2 only induced little bone formation. Together, pre-mineralization of the anisotropic SSC significantly improved the osteogenic capacity, resulting in full consolidation of the femoral defects in 4 out of 5 cases.

RhBMP–2 delivery via a collagen sponge is an FDA-approved treatment and several clinical trials have demonstrated efficacy to achieve spinal fusions, or for treatment of open tibial fractures and long bone non-unions [19]. However, several severe adverse events have been reported and the majority are believed to be a consequence of applying supra-physiological doses to the defect area [10]. Indeed, a meta-analysis indicates that the reduction of the rhBMP-2 dose correlates with a lower complication rate after performing a spinal fusion procedure [20]. Therefore, scaffold-based strategies to enhance the efficacy of rhBMP-2, and hence resulting in a reduction of the therapeutic dose required, represent an attractive modality to improve the overall safety profile.

Silk fibroin is a versatile natural polymer for scaffold production offering distinct mechanical properties when compared to other biodegradable biomaterials [12], demonstrating excellent biocompatibility as well as low immunogenicity [21]. Various silk scaffolds have been developed for bone tissue engineering, showing osteogenic potential [22]. As bone displays an aligned tissue architecture [23], scaffolds with axially aligned pores have been developed and shown to promote bone regeneration, potentially by allowing directional cell migration and ordered extracellular matrix deposition within the defect area [15,24]. However, untreated silk fibroin scaffolds generally provide poor osteoconductive properties. Coatings with bone-like apatite can yield osteoconductive scaffolds, which have been shown to provide an appropriate osteogenic environment for tissue engineering approaches [25]. A very simple and effective method for surface biomineralization which can be employed for a wide variety of scaffold materials is immersion in SBF solution, first described by Kokubo [26]. SBF contains mineral ion concentrations almost similar to those present in human blood plasma and treatment results in nanocrystalline hydroxyapatite (nHAp) which mimics bone mineral. This technique has been extensively used to coat orthopedic and dental implants, as well as polymeric and composite tissue engineering scaffolds [27]. Interestingly, SBF-treated polycaprolactone scaffolds have been shown to effectively bind growth factors, resulting in a sustained release [28]. As proteins bind to calcium phosphates [29,30,31], the rhBMP-2 loaded onto the premineralized SSC-SBF scaffold most likely was more steadily released, increasing bone regeneration.

In vitro testing revealed a decrease in ATP production of rBMSCs seeded onto the SSC-SBF scaffolds. As caspase 3/7 activity was equally lower, this is not the consequence of an increased rate of apoptosis, but potentially due to a moderately lower cell proliferation rate when the cells are seeded onto a HA-coated surface. A similar observation was made for MC3T3-E1 cells cultured on HA-coated titanium plates, where a HA-dose-dependent reduction in cell proliferation was shown [32].

Angle et al. [33] demonstrated that 12 µg of rhBMP-2 loaded onto a collagen sponge was the optimal dose to fully bridge a 5 mm CSD in the rat femur. As this and also other studies demonstrated occasional bony union after the local application of 5 µg rhBMP-2, but not with a lower dose [34], we selected a low dose of 2.5 µg in order to evaluate the performance of the SSC-SBF scaffold. Indeed, using the biomineralized biomimetic silk scaffold, delivery of 2.5 µg rhBMP-2 was sufficient to achieve bony union for 80.0% of the tested samples (4/5), indicating that the synergistic effect of nHAp and scaffold anisotropy improves BMP-2-mediated bone regeneration in vivo. However, based on the present study, it is not possible to draw a direct conclusion that the SSC-SBF scaffold was outperforming collagen as a delivery vehicle for rhBMP-2, since collagen sponges with a similar dose of BMP-2 were not included. 

Liu et al. [35] have shown that a nHA coated silk-fibroin scaffold can enhance the efficacy of BMSCs to promote bone regeneration in a rat calvarial model. Next to pre-mineralization strategies, various other scaffolds and delivery systems have been developed allowing a significant reduction of rhBMP-2. Refaat et al. [36] demonstrated that loading of a demineralized bone matrix with a combination of cartilage oligomeric matrix protein (COMP) and rhBMP-2 can reduce the effective dosage in a rat spinal fusion animal model. In the presence of COMP, the delivery of 2 μg BMP-2 achieved a similar outcome to 10 μg of BMP-2 alone. Using a similar animal model, it was shown that the implantation of a polylactic acid/polyethene glycol/nano-hydroxyapatite composite scaffold (nHAp/PLA-PEG) allowed a reduction from 10 to 3 µg of rhBMP-2 to promote efficient bone regeneration [37]. Van der Stok et al. developed porous titanium implants by selective laser melting which allowed full regeneration of 6 mm critical-sized femur defects in rats when combined with 3 µg rhBMP-2 delivered with a fibrin gel [38]. In a comparable defect model, the application of 1 µg rhBMP2 delivered via a collagen sponge infused with a combination of heparin-binding peptide amphiphiles and heparan sulfate resulted in full bridging of a 5 mm CSD in 4 out of 6 animals, whereas delivery of an equal dose via an absorbable collagen sponge did not yield any bony fusion [39]. Next to strategies delivering a single growth factor (i.e., rhBMP-2), the combinatorial application of osteogenic and angiogenic growth factors (GF) has been extensively explored to improve bone regeneration. For instance, Walsh et al. [40] demonstrated that functionalization of a highly porous, collagen–hydroxyapatite composite scaffold with 2.5 µg rhBMP-2 and 2.5 µg VEGF achieved full regeneration of a critical-sized, rat calvarial defect. Together, although various scaffolds and GF delivery systems have been developed allowing a reduction of the rhBMP-2 dose, many of these approaches are cost-intensive, not easily scalable or require multiple GFs. 

## 4. Materials and Methods

### 4.1. Fabrication of Anisotropic Silk Scaffolds and Treatment with 10× SBF

Silk fibroin (SF) solution was prepared as reported previously [41]. Briefly, SF was isolated from *Bombyx mori* silkworm cocoons. Cocoons were cut open and the silkworms disposed. After removing the inner silk cocoon layer, the material was then cut into approximately fingernail-sized pieces. Then, batches of 5 g of this cocoon material were boiled in 2 L of 20 mM sodium carbonate solution for 1 h to remove sericin. The resulting degummed fibers were thoroughly rinsed in double-distilled water (ddH_2_O) and the SF fibers were then air-dried before further processing. The degummed SF was solubilized in a 9.3 M aqueous LiBr solution at 60 °C for 3 h. Subsequently, the dissolved SF was dialyzed against ddH2O at RT using a dialysis tube with 6–8 kDa MWCO (Spectra Por, Spectrum Chemical, New Brunswick, NJ, USA) until conductivity of the dialysis water was <10 mS/cm (indicative for successful removal of LiBr). The resulting aqueous SF solution was centrifuged at 10,000 *g* for 20 min at 4 °C to remove insoluble remnants from the native cocoon. Prior to further use, the concentration of this aqueous silk solution was determined gravimetrically. The silk content was between 7.2 and 7.6% wt/v and the silk solution was stored at 4 °C up to a maximum of 3 weeks. Highly porous, anisotropic silk scaffolds were produced by controlled directional freezing of the produced SF solution [42], followed by freeze-drying and a beta-sheet re-induction step using water vapor annealing [43] followed by steam sterilization. A custom-made mold of polyoxymethylen (POM; molding area: 40 mm × 50 mm) consisting of three chambers, which are separated by two parallel-oriented metal plates with a thickness of 1 mm, was used for directional freezing. The central chamber flanked by the metal plates was filled with the SF solution. The outer chamber was filled with freezing agent, which consisted of a mixture of 20% ethanol and dry ice, resulting in a constant temperature of the metal plates of approx. −10 °C. To ensure interconnectivity of the formed directional pores and layers, a plate with polypropylene rods (spacing 3 mm; diameter 300 µm) was placed into the chamber where the SF solution was perpendicularly frozen (see Figure 1A). After ice nucleation at the cold metal plate, a stable ice front developed moving through the SF solution with a constant freezing rate. After completion of the freezing process the SF ice block was directly transferred into a lyophylizer. Through removal of the aligned ice crystals, sheet-like pores remained after freeze-drying. Beta-sheets were induced by water vapor annealing o/n at RT. Finally, the scaffolds were sterilized by autoclaving at 121 °C for 30 min. These steps induce beta-sheet formation in the SF-based scaffold rendering its insolubility in aqueous environments.

Coating of the silk scaffolds with an apatitic calcium phosphate layer was performed by treating appropriately sized scaffolds (6 mm × 4 mm × 4 mm) with 10× SBF according to a previously published protocol [44]. Briefly, pre-sized scaffolds were wetted in 100% methanol and subsequently washed and hydrated in sterile ddH_2_O for 48 h. Scaffolds were pat dried on a sterile gauze and placed into a sterile glass bottle containing 200 mL 10× SBF solution. NaHCO_3_ powder was added under constant stirring to raise the hydrogencarbonate ion (HCO3-) concentration to 10 mM. The glass bottle was tightly capped and the SSC were coated at RT for 8 h. Finally, the scaffolds were briefly washed in ddH_2_0, pat-dried, and stored in a sterile plastic pouch until further use.

Loading of the SSC with recombinant human BMP-2 (rhBMP-2; Peprotech, Vienna, Austria) was performed immediately prior to implantation into the femoral defect. Therefore, 2.5 µg rhBMP-2 in 50 µL sterile sodium chloride (NaCl) solution was loaded onto the scaffold. Control scaffolds received 50 µL NaCl solution.

### 4.2. Scanning Electron Microscopy (SEM)

For SEM, SSC were dried by a graded ethanol series (40%, 50%, 60%, 70%, 80%, 90%, 100%, 15 min each) and by increasing ethanol–hexamethyldisilazane (HMDS) series up to 100% (33%, 66%, 100%—1 h each). Subsequently, the specimens were sputter coated with Pd–Au using a Polaron SC7620 sputter coater (Quorum Technologies Ltd., East Grinstead, UK), and examined using a JEOL JSM-6510 scanning electron microscope (Jeol GmbH, Eching/Munich, Germany).

### 4.3. Isolation and Cultivation of Rat BMSCs

Rat bone marrow stromal cells (rBMSCs) were isolated as previously published [45]. Briefly, bone marrow was flushed from femurs harvested from 12 weeks old male *Sprague Dawley* rats (Janvier Labs SAS, Le Genest-Saint-Isle, France) and the flush-out solution was thoroughly resuspended in complete growth medium (αMEM supplemented with 10% FBS and 2 mM GlutaMAX^TM^) and was passed through a 70 µm cell strainer (Becton Dickinson, Vienna, Austria). Cells were then washed in PBS and were subsequently plated in complete growth medium. After 2 days in culture, non-adherent cells were removed by several washes in PBS and the adherent cells were cultured to near confluency (approx. 80%). Cells were then trypsinized and re-plated (passage 0) and were subsequently passaged at approximately 70–80% confluency. Cells at passages 2 and 3 were used for all experiments.

### 4.4. ATP and Caspase 3/7 Activity Assays

In total, 15.000 rBMSC were seeded onto each prepared scaffold (SSC and SSC-SBF) in 96-well plates and cultivated in 150 µL of complete growth medium for 3 days at 37 °C, and 5% CO_2_. After 3 days, cellular ATP content was measured as a surrogate of cell viability, using the CellTiter-Glo^®^ 3D Cell Viability Assay (Promega, Fitchburg, Wisconsin, USA) according to the manufacturer’s instructions. Caspase 3/7 activity was measured for determination of apoptotic cells, using the Caspase-Glo^®^ 3/7 Assay (Promega, Fitchburg, Wisconsin, USA) according to the manufacturer’s protocol.

### 4.5. Animal Study Design

All animal experiments and procedures were conducted in accordance with Austrian laws on animal experimentation and were approved by Austrian regulatory authorities (Permit No. BMWF-66.019/0038-V/3b/2018).

A total of 24 12 week-old, adult male *Sprague Dawley* rats (Janvier Labs SAS, France) weighing approximately 475–550 g were randomly assigned to four experimental groups of equal size (*n* = 6). A 5 mm, critical-sized, mid-femoral defect (CSD) was created (see Section 4.6). The bone defects were either treated with a silk scaffold only (Group I; SSC), a scaffold treated with 10× SBF (Group II; SSC-SBF), a SSC loaded with 2.5 µg human recombinant BMP-2 (Group III; SSC+BMP-2), or SBF-treated silk scaffold loaded with 2.5 µg hrBMP-2 (Group IV; SSC-SBF+BMP-2). For comparison, 4 animals received an untreated absorbable collagen sponge (Biopad, Euroresearch, Italy).

Radiological follow-ups in 2 planes were performed under general anesthesia 2, 6, and 10 weeks postoperatively in order to evaluate the progress of bone regeneration and the status of the osteosynthesis material. Then, 10 weeks post-surgery the animals were sacrificed and the femurs were harvested for µCT and histological analysis.

### 4.6. Surgical Procedure

A 5 mm CSD was created as previously described with minor modifications [45]. Thirty minutes preoperatively the rats received 0.03 mg/kg Buprenorphin as a subcutaneous (s.c.) injection. Anesthesia was induced in an airtight box with 4% (*v/v*) isoflurane in oxygen and subsequently was maintained at 2% (*v/v*) with a flow rate of 500 mL/min. During the entire procedure, the animals were placed on an electric heating pad to prevent hypothermia (Harvard Apparatus, Holliston, MA, USA). Prior to surgery, each animal received an antibiotic (Clindamycin, 45 mg/kg). After aseptic preparation for surgery, a 3–4 cm skin incision was made, and the shaft of the right femur was carefully exposed. Subsequently, 4 parallel drill holes were created using a 0.9 mm drill bit (Gebrüder Brasseler, Lemgo, Germany), two custom-made angle-stable fixation plates were secured to the femur, using four threaded Kirschner wires (MEDE Technik, Emmingen, Germany). A 5 mm segmental defect was created using a 0.44 mm Gigli wire saw (RISystem AG, Lanquart, Switzerland) and a custom made saw guide. Subsequently the site was thoroughly rinsed with sterile saline solution. The removed bone fragment was measured using a caliper and an appropriate SSC was press-fit into the bone defect according to the experimental treatment groups (see Section 4.2). The wound was subsequently closed in layers. Immediately after recovery from anesthesia the animals were allowed free movement. All animals received two doses of buprenorphine sc. (0.03 mg/kg, twice daily, for three days) and tramadol-hydrochloride (20 mg/kg body weight, once daily) via their drinking water for up to 5 days. The animals had free access to food and water and were frequently monitored for any complications, weight loss, or abnormal behavior. In the case of wound infection or irritation, animals were given 45 mg/kg Clindamycin s.c. Wound clips were removed after 7 days.

### 4.7. Microcomputed Tomography

The explanted femora were examined by μCT using a SCANCO micro-CT 50 system (SCANCO Medical, Brüttisellen, Switzerland). All samples were scanned nominally to the diaphyseal axis of the femur at 90 kVp and 200 μA with a 0.5 mm Al filter. In total, 500 projections/180° were integrated for 500 ms and reconstructed to an isotropic voxel size of 17.2 μm. Volume of interest (VOI) was selected using ImageJ/Fiji [46]. Image stacks were rotated so the femur was aligned with the Z axis and the fixation pins were horizontally aligned with the X axis. The bone up to 1.8 mm adjacent to the fixation pins was excluded from measurement, resulting in a VOI between 4.6 and 5.5 mm in height. The outer border of residual old bone was marked using the polygonal selection tool and interpolated over the osteotomy gap. The VOI was thresholded and exported as a mask. Measurements were performed with Definiens Developer XD 2.7 (Definiens AG, Munich, Germany). The measurement was split into a defect region, limited to a height of 4 mm centered on the VOI and a marginal region of the remaining VOI. Both regions were split into the central volume inside the interpolation of the old bone over the osteotomy gap and the surrounding volume. In all regions, Bone Volume (BV) and Tissue Volume (TV) were measured. BV/TV was calculated and is expressed as mean ± SD (%).

### 4.8. Histological Examination and Staining

The complete right femurs including muscle tissue were explanted by exarticulation at the hip and knee joint and were immediately transferred to 4% paraformaldehyde (PFA) in PBS. After 48 h at 4 °C, the samples were decalcified in 2% PFA/12.5% EDTA solution (pH = 7.5). A minimum of 3 biological replicates of each treatment group were evaluated by histology. After a minimum of 7 weeks the femora were processed for paraffin embedding and 7 μm sections were deparaffinized using Roti^®^-Histol (Carl Roth, Germany), rehydrated in a graded alcohol series and stained either with Masson–Goldner trichrome or Movat’s pentachrome stain [47]. Digital high-resolution images were acquired using a Zeiss Axioplan microscope equipped with an AxioCam MRc5 CCD camera (Carl Zeiss GmbH, Vienna, Austria).

### 4.9. Statistical Methods

For comparison of the defect bone volumes determined by µCT analysis (mean ± SD; mm^3^), a one-way ANOVA test with post-hoc pairwise comparisons (Tukey’s) was performed. For pairwise comparisons (ATP and Caspase 3/7 activity) an unpaired *t*-test was used. Samples were tested for normal distribution using the Shapiro Wilk test. Significance was set at α = 0.05. All tests were performed using GraphPad Prism v. 9.02 (San Diego, CA, USA).

## 5. Conclusions

Non-unions and large bony defects after trauma or tumor resection remain a significant clinical challenge in orthopedic surgery. The goal of treatment of such a defect is to restore bony, load-bearing capacity and to mobilize the patient as early as possible. The current gold standard usually requires one or more major surgical procedures in which bone from, e.g., the pelvis or the proximal tibia is harvested and then transplanted into the defect site. Although this procedure generally results in consolidation of the bone defect, the procedure can be lengthy and poses various risks. In particular, donor-site morbidities such as prolonged pelvic pain and iatrogenic fractures must be prevented [6]. Together, the biomimetic silk-fibroin scaffold developed in this study allows a significant reduction of rhBMP-2 dosage and thus represents a promising option for the treatment of non-unions, which are typically difficult to manage.

## Supplementary Materials

The following are available online at https://www.mdpi.com/article/10.3390/ijms23010283/s1.
