# Supplementary material for: Enhanced BMP-2-Mediated Bone Repair Using an Anisotropic Silk Fibroin Scaffold Coated with Bone-like Apatite
Source: Int J Mol Sci. 2021 Dec 28;23(1):283. doi: 10.3390/ijms23010283 (PMC8745248; doi:10.3390/ijms23010283)
Supplement: Supplementary file 1 [file ijms-23-00283-s001.zip › ijms-1491591-supplementary.pdf]

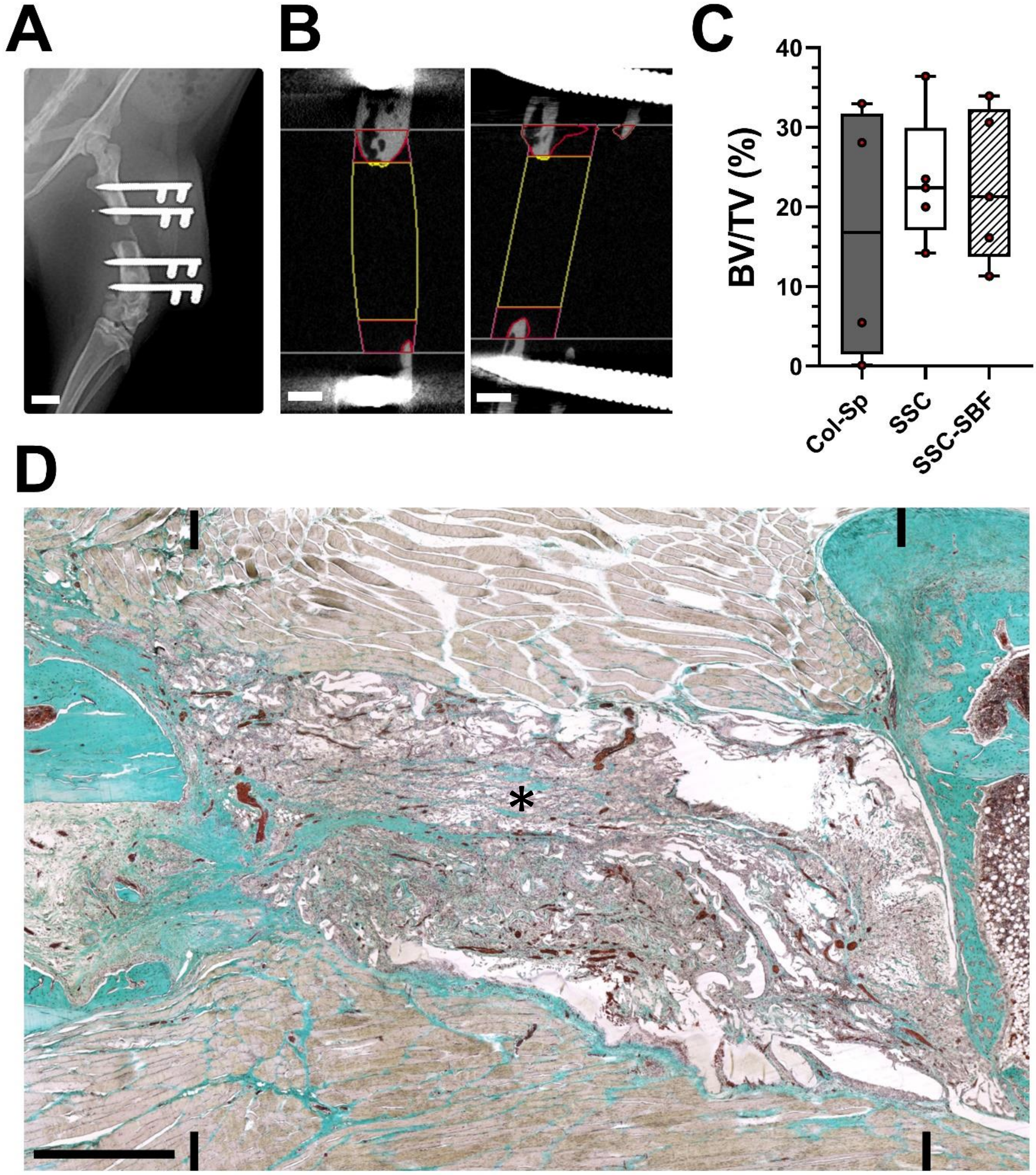

Figure S1: (**A**) X-ray (Scale bar = 4.5 mm) and (**B**) μCT images (Scale bar = 1 mm) in two planes of a representative sample after treatment with an absorbable collagen sponge only (ACS). (**C**) BV/TV measurements determined for explanted femora treated with ACS (n=4), and SSC (n=6) or SCC-SBF (n=5) for comparison (Note: the latter 2 groups are also shown in figure 5). (**D**) Masson-Goldner trichrome stained section of an ACS-treated femur, showing mostly fibrotic tissue within the defect area (indicated by *; scale bar = 1 mm).
